# Dysfibrinogenemia in Pregnancy: A Case Series Highlighting Diagnostic Challenges and Multidisciplinary Management

**DOI:** 10.7759/cureus.109384

**Published:** 2026-05-21

**Authors:** Supreet Mahurkar, Ejike Nnamani, Sarah Lewis, Sajitha Parveen

**Affiliations:** 1 Obstetrics and Gynaecology, Aneurin Bevan University Health Board, Gwent, GBR; 2 Haematology, Aneurin Bevan University Health Board, Gwent, GBR

**Keywords:** disorders of fibrinogen, dysfibrinogenemia, high risk obstetrics, low fibrinogen, pregnancy

## Abstract

Dysfibrinogenemia is a rare qualitative fibrinogen disorder associated with both bleeding and thrombotic risks, presenting unique diagnostic and management challenges in pregnancy. Physiological increases in fibrinogen during pregnancy may obscure underlying abnormalities, and reliance on functional assays alone can result in misinterpretation and inappropriate management. We present a case series of three pregnant patients in whom dysfibrinogenemia was either incidentally identified during antenatal care or recognised through prior family screening. All cases demonstrated persistently low functional fibrinogen levels measured using the Clauss method, with normal antigenic fibrinogen levels on enzyme-linked immunosorbent assay (ELISA), consistent with qualitative fibrinogen defects. Rotational thromboelastometry (ROTEM) was used to aid real‑time haemostatic assessment. Multidisciplinary management involving obstetricians, anaesthetists, and haematologists enabled individualised care, resulting in successful outcomes including spontaneous vaginal delivery, caesarean section under general anaesthesia, and medically indicated termination, without significant haemorrhagic complications. This series highlights the importance of distinguishing qualitative from quantitative fibrinogen disorders and cautions against reflexive fibrinogen replacement based solely on functional assays. A structured diagnostic approach, supported by antigen testing and viscoelastic assessment, is essential to guide safe and effective management in pregnancy.

## Introduction

Fibrinogen plays a central role in haemostasis by contributing to clot formation, platelet aggregation, and fibrinolysis [1,2]. It is an inactive precursor of fibrin and a key component in clot formation [3]. Structurally, fibrinogen comprises three pairs of disulfide‑linked polypeptide chains (Aα, Bβ, and γ), encoded on the long arm of chromosome 4 [3,4]. Fibrin assembly promotes intermolecular antiparallel alignment of γ‑chain pairs, which are subsequently cross‑linked by factor XIIIa to form γ‑dimers [2].

Congenital fibrinogen disorders may be either quantitative (hypofibrinogenemia and afibrinogenemia) or qualitative (dysfibrinogenemia) [1,4]. These uncommon conditions arise from monoallelic mutations in fibrinogen genes (FGA, FGB, and FGG) and typically follow an autosomal dominant inheritance pattern [5].

Pregnant women with congenital fibrinogen disorders are at increased risk of obstetric complications, including miscarriage, placental abruption, intraoperative bleeding, postpartum haemorrhage, impaired wound healing, and thrombosis [4]. While these complications are generally more severe in afibrinogenemia, they may also occur in hypofibrinogenemia and dysfibrinogenemia [1,6]. Management often involves fibrinogen replacement therapy, with fibrinogen concentrate increasingly utilised during pregnancy [6].

## Case presentation

Case 1

The first patient was a G2 P0+1 woman with one previous first‑trimester miscarriage. She had no history of abnormal bleeding, menorrhagia, or thrombotic events, and there was no known family history of bleeding disorders. In this index pregnancy, she received low‑dose aspirin and folic acid and had uneventful antenatal care until term. At term, she developed raised blood pressure and was assessed for pre‑eclampsia, during which routine blood investigations incidentally demonstrated a low fibrinogen level. Initial functional fibrinogen measured by the Clauss method was 0.7 g/L and remained persistently low on repeat testing at 0.8 g/L. Other coagulation parameters, including prothrombin time, activated partial thromboplastin time, and platelet count, were within normal limits. Following discussion with the haematology team, further evaluation was undertaken to assess fibrinogen antigen levels using an enzyme-linked immunosorbent assay (ELISA), which returned within the normal range. This discrepancy between functional (0.8 g/L) and antigenic ELISA fibrinogen (5.52 g/L) was consistent with a qualitative fibrinogen disorder. Baseline rotational thromboelastometry (ROTEM) demonstrated reassuring clot parameters, including a normal fibrin-based thromboelastometry (FIBTEM) A5 value (18 mm). The absence of significant bleeding following a previous miscarriage and the lack of a personal history of venous thromboembolism were considered supportive of the diagnostic impression and informed subsequent management.

A multidisciplinary delivery plan was developed involving obstetric, anaesthetic, and haematology teams. This included prophylactic administration of tranexamic acid, avoidance of neuraxial anaesthesia, and ensuring that fibrinogen concentrate was readily available in the delivery room in the event of haemorrhage.

Instrumental delivery methods, including ventouse and fetal blood sampling, were avoided due to the potential risk of an inherited bleeding disorder in the neonate. Labour was induced at term for pre‑eclampsia using Propess, and the patient subsequently achieved a vaginal delivery with tranexamic acid alone. Active management of the third stage of labour was undertaken. The measured blood loss was 366 mL, which was within normal limits. There were no maternal bleeding complications, and the neonate was born in good condition and received routine postnatal care.

Postnatally, a significant family history of venous thromboembolism in the patient’s father was identified, and extended thromboprophylaxis was prescribed for six weeks in conjunction with a short postpartum course of tranexamic acid. Genetic testing confirmed congenital dysfibrinogenemia with identification of a heterozygous pathogenic variant in the fibrinogen alpha chain gene (FGA). Subsequent family screening identified an affected first‑degree relative. The patient was counselled regarding the implications of this diagnosis for future pregnancies and invasive procedures, with a plan for tranexamic acid coverage for minor procedures and haematology input for higher‑risk interventions. She was registered for ongoing follow‑up with the haematology service.

Case 2

The second patient was a primigravida with a BMI of 38 and a history of scoliosis with previous spinal surgery. She had no personal history of excessive bleeding and had previously undergone surgical procedures without complication. She had a family history of venous thromboembolism. She received routine antenatal care, including an anaesthetic review regarding the feasibility and efficacy of neuraxial analgesia. A plan for labour analgesia using remifentanil patient‑controlled analgesia was established, with general anaesthesia planned if caesarean delivery became necessary.

She presented at term with spontaneous rupture of membranes and underwent induction of labour. During intrapartum assessment, blood tests performed for suspected chorioamnionitis revealed an incidental finding of markedly reduced functional fibrinogen. Clauss fibrinogen measured 0.9 g/L and remained persistently low on repeat testing. Fibrinogen ELISA (4.9 g/L) levels were subsequently found to be within the normal range. Other laboratory parameters, including full blood count, platelet count, and liver function tests, were within normal limits. There was no clinical or laboratory evidence of consumptive coagulopathy.

The case was reviewed urgently by the haematology team. In the context of persistently low functional fibrinogen, normal antigenic fibrinogen levels, prior uneventful surgical history, and a family history of thrombosis, a hereditary qualitative fibrinogen disorder was suspected. Following confirmation of normal fibrinogen ELISA levels (4.9 g/L), a diagnosis of dysfibrinogenemia was made.

In anticipation of delivery outside standard working hours, and prior to confirmation of the diagnosis, while awaiting fibrinogen ELISA results, tranexamic acid and fibrinogen concentrate were administered following multidisciplinary discussion. A dose of 2 g fibrinogen concentrate was given at the induction of general anaesthesia, based on a multidisciplinary risk-benefit assessment balancing bleeding risk against potential thrombotic complications. Due to failure to progress in labour and concerns regarding intrauterine infection, caesarean delivery was performed under general anaesthesia. Intraoperative haemostasis was satisfactory. The measured blood loss was 875 mL, and 220 mL of salvaged blood was returned using cell salvage. A Bakri balloon and vaginal pack were placed intraoperatively and subsequently removed without complication.

Postoperatively, functional fibrinogen remained low at 0.8 g/L, and no further fibrinogen replacement was required. The patient experienced an uncomplicated postoperative recovery and remained an inpatient for eight days. In view of the underlying fibrinogen disorder and family history of venous thromboembolism, extended postpartum thromboprophylaxis with low‑molecular‑weight heparin was commenced for six weeks.

Subsequent genetic testing confirmed congenital dysfibrinogenemia due to a heterozygous pathogenic variant in the fibrinogen alpha chain gene (FGA). Family members were offered screening, and the patient was referred for long‑term haematology follow‑up.

Case 3

The third patient had congenital dysfibrinogenemia, diagnosed through family screening after her mother was diagnosed with the condition. Despite this diagnosis, she had remained asymptomatic for many years and reported no bleeding complications during prior surgical interventions. As a result of her prolonged asymptomatic course, the diagnosis was not initially considered clinically relevant by the patient and was not recalled at the time of presentation.

She presented during her first pregnancy for medical termination at 24 weeks’ gestation due to a fetal cardiac anomaly. Pre‑procedure laboratory investigations demonstrated a markedly reduced functional (Clauss) fibrinogen level of 0.5 g/L. Further evaluation revealed normal fibrinogen ELISA levels (4.08 g/L). Review of historical medical records and discussion with the haematology team confirmed her prior diagnosis of congenital dysfibrinogenemia, consistent with the observed discrepancy between functional and antigenic fibrinogen assays. ROTEM demonstrated reduced fibrin‑dependent clot strength, with a FIBTEM A5 value of 11 mm.

Given the anticipated haemostatic challenges associated with intrauterine fetal demise, underlying dysfibrinogenemia, and reduced fibrin contribution on ROTEM, a multidisciplinary management plan was implemented. Tranexamic acid was commenced, and fibrinogen concentrate (2 g) was administered prior to delivery. Additional fibrinogen concentrate was made available in the delivery room if required. Neuraxial anaesthesia was avoided, and analgesia was provided using patient‑controlled analgesia.

The termination procedure was completed without significant haemorrhage, with a measured blood loss of approximately 100 mL. Postpartum haemostasis remained satisfactory, and no further fibrinogen replacement was required. In view of the underlying fibrinogen disorder and a family history of thrombosis, six weeks postpartum, low molecular weight heparin (LMWH) thromboprophylaxis was prescribed.

The patient received further counselling regarding the implications of dysfibrinogenemia, including the importance of communicating the diagnosis during future healthcare encounters and pregnancy planning. Arrangements were made for continued follow‑up under the haematology service.

To facilitate comparison across cases and improve clarity of presentation, a summary of the key clinical characteristics, laboratory findings, management strategies, and outcomes is provided in Table 1. This provides a structured overview of the similarities and differences between cases, particularly in relation to diagnostic findings, multidisciplinary decision‑making, and haemostatic management, thereby reinforcing the key themes highlighted in this series.

**Table 1:** Summary of clinical characteristics, investigations, management, and outcomes PET: preeclampsia; G2P0+1: gravida 2, para 0+1; ELISA: enzyme-linked immunosorbent assay; CS: caesarean section; GA: general anaesthesia; TXA: tranexamic acid; LMWH: low-molecular-weight heparin; FGA: fibrinogen alpha chain gene

Feature	Case 1	Case 2	Case 3
Presentation	Incidental finding during PET workup	Incidental finding intrapartum (chorioamnionitis suspicion)	Known dysfibrinogenemia (family screening)
Parity	G2P0+1	Primigravida	Not specified
Clauss fibrinogen	0.7–0.8 g/L	0.8–0.9 g/L	0.5 g/L
ELISA fibrinogen	5.52 g/L	4.9 g/L	4.08 g/L
Diagnosis	Dysfibrinogenemia (confirmed genetically)	Dysfibrinogenemia (confirmed genetically)	Known dysfibrinogenemia
Mode of delivery	Vaginal delivery	CS under GA	Medical termination (24 wks)
Haemostatic management	TXA only	TXA + fibrinogen concentrate (2 g)	TXA + fibrinogen concentrate (2 g pre‑procedure)
Blood loss	366 mL	875 mL	~100 mL
Complications	None	None	None
Thromboprophylaxis	6 weeks LMWH	6 weeks LMWH	6 weeks LMWH
Genetics	FGA mutation	FGA mutation	Known condition

## Discussion

Pregnancy in Women with congenital dysfibrinogenemia is classified as high-risk. Though most of the pregnancies run asymptomatic courses, there has been documented evidence of complications including miscarriages, stillbirth, abruptio placentae, fetal growth restriction, postpartum haemorrhage and thrombosis [5,7]. 

Physiologically, fibrinogen levels increase throughout pregnancy, and therefore any reduction below the normal range should be regarded as abnormal and prompt further investigation [8]. Failure to recognise and appropriately evaluate low fibrinogen levels may place pregnant women at increased risk of haemorrhage, inappropriate intervention, or unrecognised thrombotic complications.

In clinical practice, the differential diagnosis of low fibrinogen in pregnancy is broad and includes both acquired and inherited causes. Acquired causes such as liver dysfunction, sepsis, disseminated intravascular coagulation, and massive haemorrhage should be considered first and can often be excluded through clinical assessment and routine laboratory investigations [9,10].

 In the absence of an obvious acquired cause, inherited fibrinogen disorders should be actively considered. Importantly, functional fibrinogen measured by the Clauss method does not distinguish between quantitative deficiencies, such as hypofibrinogenemia, and qualitative disorders, such as dysfibrinogenemia [11]. Measurement of antigenic fibrinogen ELISA is therefore crucial, as a discrepancy between reduced functional fibrinogen and normal ELISA antigen levels strongly suggests dysfibrinogenemia [12]. Recognition of this distinction has significant implications for clinical management, as indiscriminate fibrinogen replacement may not always be appropriate and may increase thrombotic risk in certain fibrinogen disorders.

The cases presented highlight the importance of a structured diagnostic approach when low fibrinogen is identified. Rather than intuitively correcting a low Clauss fibrinogen value, it is essential to interpret results within the broader clinical and haematological context. In situations where antigenic fibrinogen ELISA assays are not immediately available, ROTEM can provide rapid, point‑of‑care assessment of overall haemostasis [13]. ROTEM offers valuable information regarding clot formation and fibrin contribution and was used in our cases to support real‑time clinical decision‑making [13,14]. However, ROTEM should be interpreted cautiously, as reassuring parameters may be present despite markedly reduced functional fibrinogen, particularly in qualitative fibrinogen disorders. As such, ROTEM should complement, rather than replace, standard laboratory investigations [15].

Management of low fibrinogen in pregnancy requires multidisciplinary team involvement, typically including obstetricians, anaesthetists, haematologists, neonatologists, pharmacists, and midwives [5]. Anaesthetic considerations are particularly important, with neuraxial techniques often avoided due to concerns regarding bleeding risk [16]. Obstetric management may involve avoidance of invasive fetal monitoring, instrumental delivery, and fetal blood sampling where an inherited bleeding disorder is suspected [17]. Haematology input is essential to support balanced decision‑making around fibrinogen replacement and thromboprophylaxis, particularly given the paradoxical association between dysfibrinogenemia and both bleeding and thrombotic risk [18]. Our cases demonstrated that individualised management plans can safely support a range of clinical outcomes, including spontaneous vaginal delivery, caesarean section, and medically indicated termination of pregnancy.

A further crucial learning point highlighted by this series is that inherited fibrinogen disorders may be forgotten or under‑recognised in asymptomatic individuals, particularly when diagnoses are made through family screening at a young age, as occurred in our case three. This has significant implications for pregnancy and surgical care later in life. Effective communication with pregnant women is therefore central to safe management, especially when diagnostic uncertainty remains while awaiting confirmatory testing. Explaining the dual risks of bleeding and thrombosis can be challenging, and midwives play a critical role in supporting shared decision‑making, reassurance, and continuity of care.

Broadly, this case series emphasises the importance of maintaining a high index of suspicion when low fibrinogen is identified during pregnancy and adopting a thoughtful, stepwise approach to diagnosis and management. Early multidisciplinary involvement and careful interpretation of laboratory and viscoelastic testing can facilitate safe and individualised care for women with both known and newly diagnosed fibrinogen disorders to avoid the use of unneeded medications that could precipitate the undesired complications associated with this condition.

## Conclusions

Congenital dysfibrinogenemia, though a rare disorder, poses a particular challenge in its management and thus, a multidisciplinary approach involving the maternal medicine specialists, haematologists, anaesthetists, midwives, pharmacists and blood bank services must be encouraged promptly.

Because fibrinogen levels normally rise during pregnancy, any low value should prompt thorough assessments of the clinical and laboratory parameters to guide treatment, instead of reflexive replacement of presumed low factors. Distinguishing qualitative from quantitative fibrinogen disorders using both functional and antigenic assays is critical, as this distinction has important implications for bleeding risk, thrombosis, and peripartum management. A structured diagnostic approach, supported by multidisciplinary collaboration, allows safe individualised patient care. This case series aimed to contribute to the limited literature on dysfibrinogenemia in pregnancy and highlights the importance of a multidisciplinary approach in its management.
