# Correction: Dysfibrinogenemia in Pregnancy: A Case Series Highlighting Diagnostic Challenges and Multidisciplinary Management

**DOI:** 10.7759/cureus.c436

**Published:** 2026-05-22

**Authors:** Supreet Mahurkar, Ejike Nnamani, Sarah Lewis, Sajitha Parveen

**Affiliations:** 1 Obstetrics and Gynaecology, Aneurin Bevan University Health Board, Gwent, GBR; 2 Haematology, Aneurin Bevan University Health Board, Gwent, GBR

The legend for Table 1 has been corrected to indicate that "PET" is short for preeclampsia.

